# Estimating double burden of malnutrition among rural and urban children in Amazonia using Bayesian latent models

**DOI:** 10.3389/fpubh.2025.1481397

**Published:** 2025-03-12

**Authors:** Jesem Douglas Yamall Orellana, Luke Parry, Francine Silva Dos Santos, Laísa Rodrigues Moreira, Patricia Carignano Torres, Antônio Alcirley da Silva Balieiro, Fernanda Rodrigues Fonseca, Paula Moraga, Erick Albacharro Chacón-Montalván

**Affiliations:** ^1^Instituto Leônidas & Maria Deane (ILMD/Fiocruz Amazônia), Manaus, Amazonas, Brazil; ^2^Lancaster Environment Centre, Lancaster University, Lancaster, United Kingdom; ^3^Instituto Amazônico de Agriculturas Familiares, Federal University of Pará, Belém, Pará, Brazil; ^4^Department of Nutrition, Federal University of Porto Alegre, Health Sciences, Porto Alegre, Rio de Janeiro, Brazil; ^5^Municipal Social Assistance Secretariat (SEMAS), Florianópolis, Brazil; ^6^Graduate Program in Complex Systems Modeling, School of Arts, Sciences and Humanities, University of São Paulo, São Paulo, Brazil; ^7^Division of Computer, Electrical and Mathematical Science and Engineering, King Abdullah University of Science and Technology, Makkah, Saudi Arabia

**Keywords:** child malnutrition, health transition, Latin America, Bayesian, epidemiological, hard to reach areas

## Abstract

**Background:**

The double burden of malnutrition (DBM) in the same individual is a neglected public health concern, especially in low- and middle-income countries (LMICs). The DBM is associated with increased risks of non-communicable diseases, childbirth complications, and healthcare costs related to obesity in adulthood. However, evaluating low prevalence outcomes in relatively small populations is challenging using conventional frequentist statistics. Our study used Bayesian latent models to estimate DBM prevalence at the individual-level in small populations located in remote towns and rural communities in the Brazilian Amazon.

**Methods:**

We employed a cross-sectional survey of urban and rural children aged 6–59 months, considering DBM as the coexistence of stunting and overweight in the same individual. We evaluated four river-dependent municipalities, sampling children in randomly selected households in each town and a total of 60 riverine forest-proximate communities. Through Bayesian modeling we estimated the latent double burden of malnutrition (LDBM) and credible intervals (CI).

**Results:**

The exceedance probability of LDBM was used to quantify this form of malnutrition at the population level. Rural prevalence of LDBM was significantly higher in Jutai (3.3%; CI: 1.5% to 6.7%) compared to Maues and Caapiranga. The likelihood that LDBM rural prevalence exceeded 1% was very high in Jutai (99.7%), and Ipixuna (63.2%), and very low (< 2%) in rural communities elsewhere. Exceedance probabilities (at 1%) also varied widely among urban sub-populations, from 6.7% in Maues to 41.2% in Caapiranga. The exceedance probability of LDBM prevalence being above 3.0% was high in rural Jutai (59.7%).

**Discussion:**

Our results have important implications for assessing DBM in vulnerable and marginalized populations, where health and nutritional status are often poorest, and public health efforts remain focused on undernutrition. Our analytical approach could enable more accurate estimation of low prevalence health outcomes, and strengthen DBM monitoring of hard-to-reach populations.

## Introduction

The co-occurrence of undernutrition and overweight is known as the double burden of malnutrition (DBM), an emerging health concern which is characterized by a rapid increase in the prevalence of overweight individuals and slow reduction in rates of undernutrition (1, 2), particularly in low- and middle-income countries (LMICs) (3–7). DBM is shaped by changing diets and physical activity patterns, and is associated with increased risks of non-communicable diseases, childbirth complications, and elevated health costs related to obesity in adulthood (8, 9).

DBM can be assessed at the level of population (e.g., country, sub-national region, or rural community), household, and individual. Around nine-in-ten studies have estimated DBM prevalence at the community/population level (1, 7, 10), largely because this aggregation facilitates straightforward comparison among different populations. Estimating the prevalence of stunting and overweight DBM at the individual level in remote towns and rural communities can be challenging in LMICS, where child health surveys tend to have limited coverage and sampling biased toward larger cities, often excluding vulnerable populations such as indigenous people and other traditional rural populations (11). Reliable prevalence estimates are also difficult when studies have relatively small, statistically underpowered sample sizes (12, 13). Nonetheless, reliable estimates of malnutrition are essential for assessing the scale of nutritional problems in specific contexts, and guiding related interventions by state and non-state institutions (14).

In preschool children, estimates of stunting and overweight DBM prevalence at the individual level are low (typically below 3%), and are mostly based on samples from country-scale or sub-national regional scales (5, 7), in which severe spatial and social inequities in health determinants become homogenized. A lack of stunting and overweight DBM research for specific geographies (including separating rural and urban sub-populations) and vulnerable populations (e.g., traditional forest-dwelling peoples in Amazonia and elsewhere) contributes to poor understanding of this health problem in LMICs (5, 15, 16).

Nutritional epidemiological studies typically characterize malnutrition based on observed anthropometric values (e.g., height-for-age z-scores) which fall above or below predefined cutoff points (e.g., 2 standard deviations below the reference population's median value). Interpreting prognostic risk is problematic for values near thresholds, near marginal values, and for ethnic minorities (17, 18). Given generally low prevalence of DBM at the individual level when using thresholds recommended by the World Health Organization (WHO), some authors adopt alternative definitions [e.g., Sagastume et al. (7) identified 17 DBM typologies] or even alternative thresholds, mainly for the overweight indicator (19, 20), hindering comparison across studies. Furthermore, for research into specific populations and/or with relatively small sample sizes, point estimates and interval estimates based on frequentist statistics can be inappropriate due to the likelihood of Type 2 error (false negatives, leading to under-estimation of DBM) and unfeasible for low prevalence nutritional outcomes.

Overcoming the challenges in estimating low prevalence nutritional outcomes in specific populations with restricted sample sizes is necessary for effectively monitoring DBM in LMICs, including robustly evaluating potential interventions for vulnerable populations. In this study, we will use the latent risk of double burden of malnutrition (LDBM) to estimate the magnitude of a low prevalence outcome. In doing so, we attempt to overcome the limitations of conventional frequentist approaches for estimating DBM prevalence, particularly for relatively small populations where the health and nutrition indexes are often poorest (21–24), or situations in which obtaining anthropometric measurements from thousands of individuals is not practical.

Here, we define LDBM as the probability of stunting and overweight co-occurring. We use “latent” to describe the risk or probability of stunting and overweight occurrence because we do not directly observe and calculate this value, due to unknown population parameters θ. The population parameters capture important characteristics associated with the outcome, such as mean, variance, and correlation. However, this probability can be estimated through the joint modeling of the two nutritional outcomes of interest, and the associated uncertainty can be quantified by generalizing the uncertainty arising from θ, a task well-suited to Bayesian inference. The LDBM definition allows estimation even with few observed cases of DBM, since this probability can be extracted from the properties of the joint density rather than a proportion of observed cases. Hence, we consider a bivariate vector **Y** = (*Y*^(1)^, *Y*^(2)^) consisting of two variables related to health outcomes, *Y*^(1)^ and *Y*^(2)^ (e.g., height/length-for-age index and body mass index), characterized by a joint density function *f*_θ_(*y*_1_, *y*_2_) that depends on population parameters θ. Given θ, LDBM is defined as $\mathrm{Pr}\left(Y^{\left(1\right)}<t_{1},Y^{\left(2\right)}>t_{2}|\theta \;\right)$.

Specifically, this paper draws on a unique dataset of rural and urban children in four remote, river-dependent municipalities in the Brazilian Amazon to examine whether latent Bayesian models may enable researchers to estimate DBM with modest sample sizes. We estimate LDBM as the probability of encountering two kinds of malnutrition (stunting and overweight) in the same individual child, selected randomly from towns and rural communities.

## Methods

### Study design

**Figure 1:**
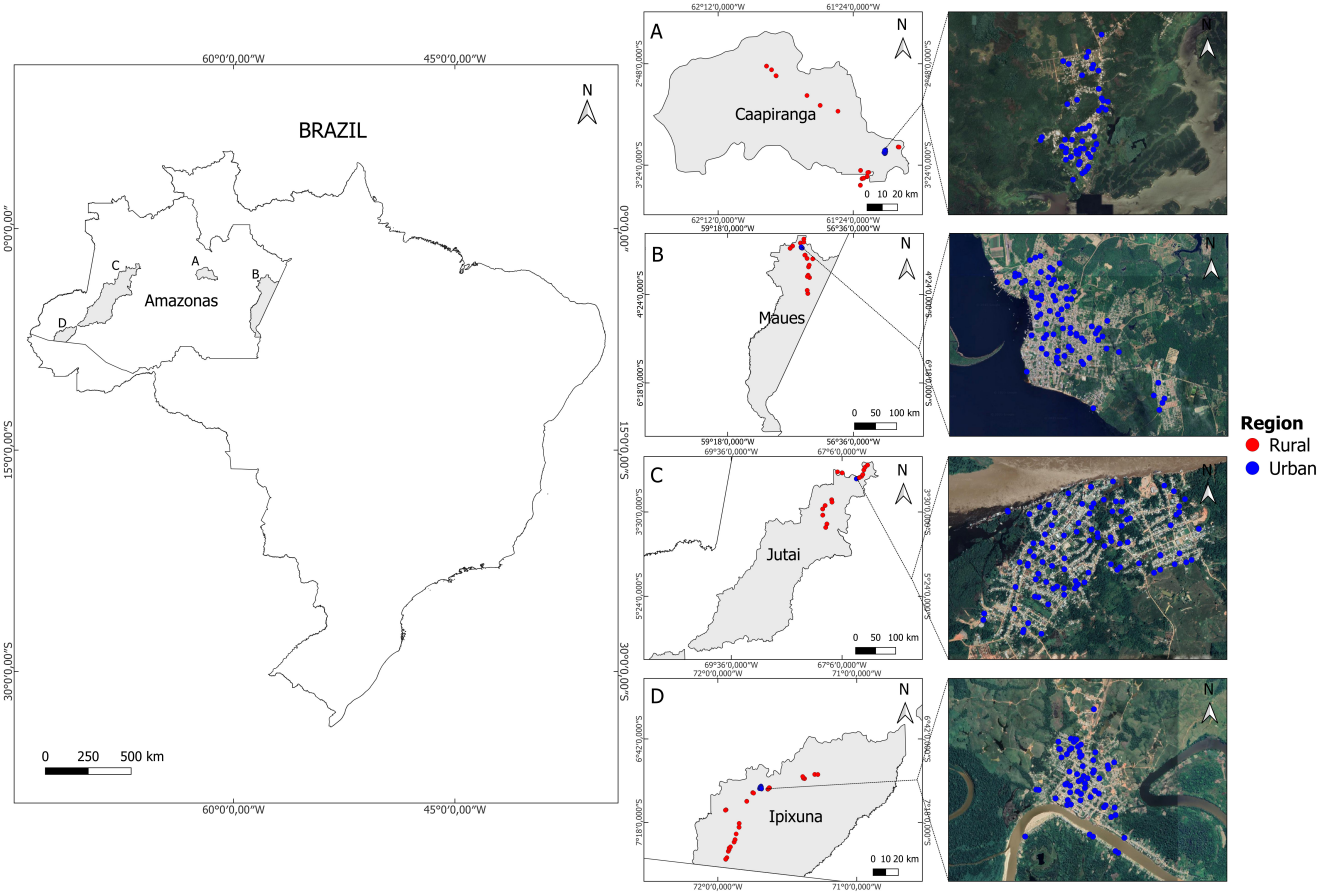
Map of the study area constituting four highly-forested river-dependent municipalities in Amazonas State, Brazil. In each municipality [**(A-D)** where gray shading indicates the municipality's territory], we sampled children within randomly selected households in the town, and surrounding rural settlements.

We conducted a cross-sectional, population-based study in 2015 and 2016 in four municipalities (Caapiranga, Ipixuna, Jutai, Maues; each composed of an urban center of the same name, and a surrounding rural area) in Amazonas State, Brazil (Figure 1). The selected municipalities were all highly river-dependent and their urban centers have relatively high social vulnerability (e.g., high income poverty and inequality, and deficiencies in terms of household access to tapped water and sanitation, educational continuity, and primary healthcare) relative to urban centers that are road-connected and/or closer to major cities within Amazonia's hierarchical urban network (25). The municipalities were all highly-forested with >90% of their original forest cover remaining, at the time. Within the study “universe” of river-dependent municipalities, the four we selected were purposefully far from each other, with varied remoteness from major cities. This remoteness shapes access to markets, and public and private institutions (e.g., universities, hospitals). Travel distance by boat from the state capital, Manaus, ranged from 162-km (Caapiranga), 342-km (Maues), 947-km (Jutai), to 2,566-km (Ipixuna). Maues was medium-sized (c. 35,000 residents) and the other towns were small (< 15,000 urban residents) (26).

In order to compare LDBM across rural and urban sub-populations, we sampled children under-5-years-old in randomly selected households in each town (i.e., the urban center of that municipality) and 60 riverine forest communities, in total (Figures 1A–D) (27). Surveyed households were selected in the context of a broader research project, investigating child health (27) and household food insecurity (28). Consequently, sampling included some households in which there were no children under-5-years-old. In each town, 200 households were randomly sampled as part of the broader study. Accordingly, 200 urban locations were randomly generated within the boundaries of each town (i.e., 800 urban households across the four towns). Urban sampling density was corrected for population density based on census sector-level information from the official 2010 demographic census (25). Urban sampling points were generated using ArcGIS 10.3 along the streets (within 20 meters) and were restricted to the potentially habitable area (using satellite imagery and openstreetmap.org). For each municipality, we intended to sample 80 rural households from 16 surrounding rural communities (five households per community, totaling 320 households from 64 communities) but the final sample was 311 households from 63 communities (27). These communities were not randomly selected but instead chosen because they covered diverse geographies, including: a gradient in travel distance from the nearest town (7–249 km); locations inside and outside of Sustainable Use Reserves; locations on the main Amazon channel, and second- and third-order tributaries; flooded-forest (*várzea*) and non-floodplain (*terra firme*) contexts.

Within each community, we first worked with residents to develop a list of all inhabited households, and then from these we randomly selected five households, whom we invited to participate in the study. In this paper, we only include data from those urban and rural households with children under-5-years-old. All children aged 6-to-59 months residing in each household were considered eligible (i.e., we did not have an expected number of children in the planned household sample but instead sampled all eligible children within sampled households). The sample for this paper comprises 422 households (all georeferenced) and 585 children (Figure 1), predominantly urban (67.1% of households *n* = 283); 65.0% of sampled children (*n* = 380; Table 1). Reflecting municipality-scale demographic differences and greater household sampling effort in towns, the number of sampled children was smallest in Caapiranga (urban = 65; rural = 35), and largest in Jutai (urban = 131; rural = 74) (Table 1).

**Table 1:** Characteristics of children under 60 months-old and households evaluated, according to area and municipality, Amazonas, Brazil, 2015–16.

**Area**	**Municipality**	**Children**	**Households**	**Children per household**	**DBM**
Rural	Caapiranga	35	27	1.3	0%
Rural	Maues	44	33	1.3	0%
Rural	Jutai	74	42	1.8	4.1%
Rural	Ipixuna	52	37	1.4	1.9%
Urban	Caapiranga	65	50	1.3	1.5%
Urban	Maues	108	80	1.4	1.9%
Urban	Jutai	131	91	1.4	0.8%
Urban	Ipixuna	76	62	1.2	0%

#### Data collection and key variables

We used a structured questionnaire which was piloted beforehand in another municipality (Autazes) in Amazonas State, with similar geographic characteristics to the four described above. This paper draws on questions in the socio-demographic and child health sections of the survey instrument. When possible, childbirth dates were obtained from official documents held by caregivers. The field research team spent 1 week training in standardized anthropometric data-collection techniques in Manaus, prior to starting fieldwork. In each municipality, half of the urban and rural sample was collected during a low-water dry season field campaign (3–4 weeks per campaign, between August–December 2015) and half during a high-water wet season campaign (March–July 2016). Each household visit was carried out by a pair of experienced, trained interviewers. Each field campaign included a team of six researchers (i.e., 3 pairs), four of whom were involved in all field campaigns. The other two team members switched halfway through, with further training provided for the two new team members. All researchers were Brazilian, with Masters-level education or above. All anthropometric data collection followed (29, 30) under supervision of the first author. Weight and length/height measurements were collected twice for the same individual, and the average value was used in the analyses. The z-scores of the height-for-age and the Body Mass Index BMI-for-age indicators were estimated from the growth curves of the WHO (29). Height-for-age z-scores below −2 were considered indicative of stunting. Z-scores above 2 for BMI-for-age were considered indicators of overweight individuals. Height-for-age z-scores below −6 or above 6, and BMI-for-age z-scores below −5 or above 5 were considered implausible (29). To reiterate, we assessed DBM at the individual level, defined by the co-occurrence of stunting and overweight (31), a conventional indicator to estimate the child DBM at the individual level (32, 33).

#### Ethics

Data collection was approved by the Brazilian Health Ethics Commission (*Comissão Nacional de Ética em Pesquisa do Conselho Nacional de Saúde*, Protocol 45383215.5.0000.0005) and Lancaster University's Research Ethics Committee (S2014/126). Anonymity, voluntary participation and other ethical considerations were ensured at all stages of the research.

#### Code availability

Our analysis of the latent double burden of malnutrition was performed using the Julia programming language (34). All the code for our analysis, including data cleaning and processing, exploratory data analysis, modeling, and summarizing results, is available at https://erickchacon.gitlab.io/latent-double-burden.

### Statistical analysis

#### Bayesian model

The latent double burden of malnutrition (LDBM), which refers to the probability of encountering two malnutrition outcomes in the same person (in this case, child) randomly selected from a population, can be expressed by the following equation:


(1) $$\begin{array}{l} p=\mathrm{Pr}\left(Y^{\left(1\right)}<t_{1},Y^{\left(2\right)}>t_{2}|\theta \right), \end{array}$$

where the problem of malnutrition occurs if the height-for-age z-scores, *Y*^(1)^, is lower than *t*_1_ and the BMI-for-age, *Y*^(2)^, is greater than *t*_2_. The comparison direction and thresholds can be easily modified if using other health variables. We modeled LDBM in the four municipalities (*i* = 1, 2, 3, 4), distinguishing between municipal samples from rural areas (*j* = 1), and urban centers (*j* = 2). It is assumed that the *k*-th pair of observations $y_{ijk}=\left(y_{ijk}^{\left(1\right)},y_{ijk}^{\left(2\right)}\right)$ in region *j* of municipality *i* comes from a bivariate normal distribution:


(2) $$\begin{array}{l} Y_{ijk}\sim MVN\left(\mu_{ij},\Sigma_{ij}\right). \end{array}$$

Here, $\mu_{ij}=\left(\mu_{ij}^{\left(1\right)},\mu_{ij}^{\left(2\right)}\right)$ and Σ_*ij*_ are the mean vector and 2 × 2 covariance matrix for the health outcome variables in region type *j* of municipality *i* . The covariance matrix is parametrized with variances ${\left(\sigma_{ij}^{\left(1\right)}\right)}^{2}$ and ${\left(\sigma_{ij}^{\left(2\right)}\right)}^{2}$ on the diagonal, and the covariance $\rho_{ij}\times \sigma_{ij}^{\left(1\right)}\times \sigma_{ij}^{\left(2\right)}$ on the off-diagonal. Notice that $\sigma_{ij}^{\left(1\right)}$and $\sigma_{ij}^{\left(2\right)}$ are the standard deviations of the nutrition indicator variables in rural and rural areas of a municipality, and ρ_*i, j*_ represents the correlation between these variables. It is assumed that the three parameters vary between different municipalities due to differences in their determinants of health and nutrition. Depending on the application, other assumptions may be made, including the assumption of constant correlation across municipalities. Therefore, these parameters need to be estimated in order to then calculate the LDBM.

The formulation of our Bayesian model was completed by defining the prior distribution for the parameters. We assumed flat uninformative priors for the mean parameters, $\pi \left(\mu_{ij}^{\left(l\right)}\right)\propto 1$ for *l* = 1, 2 . Furthermore, a uniform prior was defined for the correlation parameter, ρ_*ij*_~*U*(−1, 1), and log-flat priors were assumed for the standard deviation parameters, $\pi \left(\log\left(\sigma_{ij}^{\left(l\right)}\right)\right)\propto 1$ for *l* = 1, 2.

Bayesian inference is achieved by calculating the posterior distribution of the parameters, π(μ_*ij*_, Σ_*ij*_|*y*_*ij*1_, *y*_*ij*2_, ⋯ , *y*_*ij*_*n*__*ij*__), or by obtaining samples from this distribution. We used the Hamiltonian Monte Carlo (HMC) method to obtain samples $\mu_{ij}^{\left[m\right]}$ and $\Sigma_{ij}^{\left[m\right]}$ for *m* = 1, ⋯ , *M* , where *M* represents the total number of stored samples. The Turing.jl package in the Julia programming language was used for this purpose (35).

#### Predicting LDBM

The exceedance probability, used to quantify the magnitude of malnutrition at the population level, was estimated using the posterior LDBM, which is the probability distribution of LDBM given the set of observed values in our sample. It is defined as:


$$\begin{array}{l} \pi \left(p_{ij}|y_{ij1},\cdots ,y_{ijn_{ij}}\right)=\int \pi \left(p_{ij,}\mu_{ij,}\Sigma_{ij}|y_{ij1},\cdots ,y_{ijn_{ij}}\right)d\mu_{ij}d\Sigma_{ij}\\ \pi \left(p_{ij}|y_{ij1},\cdots ,y_{ijn_{ij}}\right)=\int \pi \left(\mu_{ij,}\Sigma_{ij}|y_{ij1},\cdots ,y_{ijn_{ij}}\right)\\ \;\pi \left(p_{ij}|\mu_{ij,}\Sigma_{ij}\right)d\mu_{ij}d\Sigma_{ij} \end{array}$$

where the first term of the integral is the posterior distribution of the parameters, and the second term is the probability of LDBM, based on some observed values for the parameters. Samples from this posterior distribution of LDBM in rural and urban regions *j* of municipality *i* are obtained using samples $\mu_{ij}^{\left[m\right]}$ and $\Sigma_{ij}^{\left[m\right]}$ for *m* = 1, ⋯ , *M* from the posterior distribution and calculating:


(3) $$\begin{array}{l} p_{ij}^{\left[m\right]}=\mathrm{Pr}\left(Y_{ij}^{\left(1\right)}>t_{1},Y_{ij}^{\left(2\right)}<t_{2}|\mu_{ij}^{\left[m\right]},\Sigma_{ij}^{\left[m\right]}\right). \end{array}$$

This can be done using the properties of a bivariate normal distribution and can also be calculated when the outcomes are jointly above or below certain cutoff points. The resulting collection $\left(p_{ij}^{\left[1\right]},p_{ij}^{\left[2\right]},\cdots \;,p_{ij}^{\left[M\right]}\right)$ consisted of 20,000 realizations from the posterior distribution of LDBM that can be used to provide point estimates and their respective credible intervals, as well as exceedance probabilities of the outcome under different circumstances (prevalences being 1%; 3% or correlation 0; see Results section).

#### Considering association between individuals

In the analysis of the double burden on children, some individuals may belong to the same household, leading to potential associations due to shared exposure factors. To account for this, we can extend the previously presented model by incorporating a bivariate household-level random effect, *W*_*h*_. Let *Y*_*hk*_ represent the health outcomes of the *k*-th child in household *h*, then the conditional distribution can be defined as *Y*_*hk*_|*W*_*h*_~*MVN*(μ+*W*_*h*_, Σ_*ij*_), such as children from the same household share the common random effect *W*_*h*_. To ensure identifiability, we assume a zero-mean Gaussian distribution for the bivariate random effect, *W*_*h*_~*MVN*(0, Σ_*w*_), with diagonal covariance matrix Σ_*w*_. Note that if household dependency exists only for one health outcome, *W*_*h*_ can be unidimensional. The resulting marginal distribution of the bivariate health outcome is *Y*_*hk*_~*MVN*(μ, Σ_*w*_+Σ). Using this distribution, the LDBM is computed as explained in the previous section (Equation 3).

## Results

We analyzed the LDBM in rural and urban areas of four municipalities. Given that some children belonged to the same households (Table 1), we first assessed the need to account for household-level dependency. Using likelihood ratio tests and comparing the Akaike Information Criteria (AIC) for z-scores of height-for-age and BMI-for-age, we found no significant improvement from adding random effects in most municipalities and area types at a 10% significance level. However, significant improvements were observed for the urban sub-population in Jutai when including random effects for height-for-age and for the urban sub-population in Ipixuna for BMI-for-age (Supplementary Table 1). Consequently, we applied the LDBM model with household-level random effects for height-for-age in urban Jutai, for BMI-for-age in urban Ipixuna, and without random effects for all other sub-populations. Models with random effects were re-parametrized after inference to ensure comparability with models without random effects.

**Figure 2:**
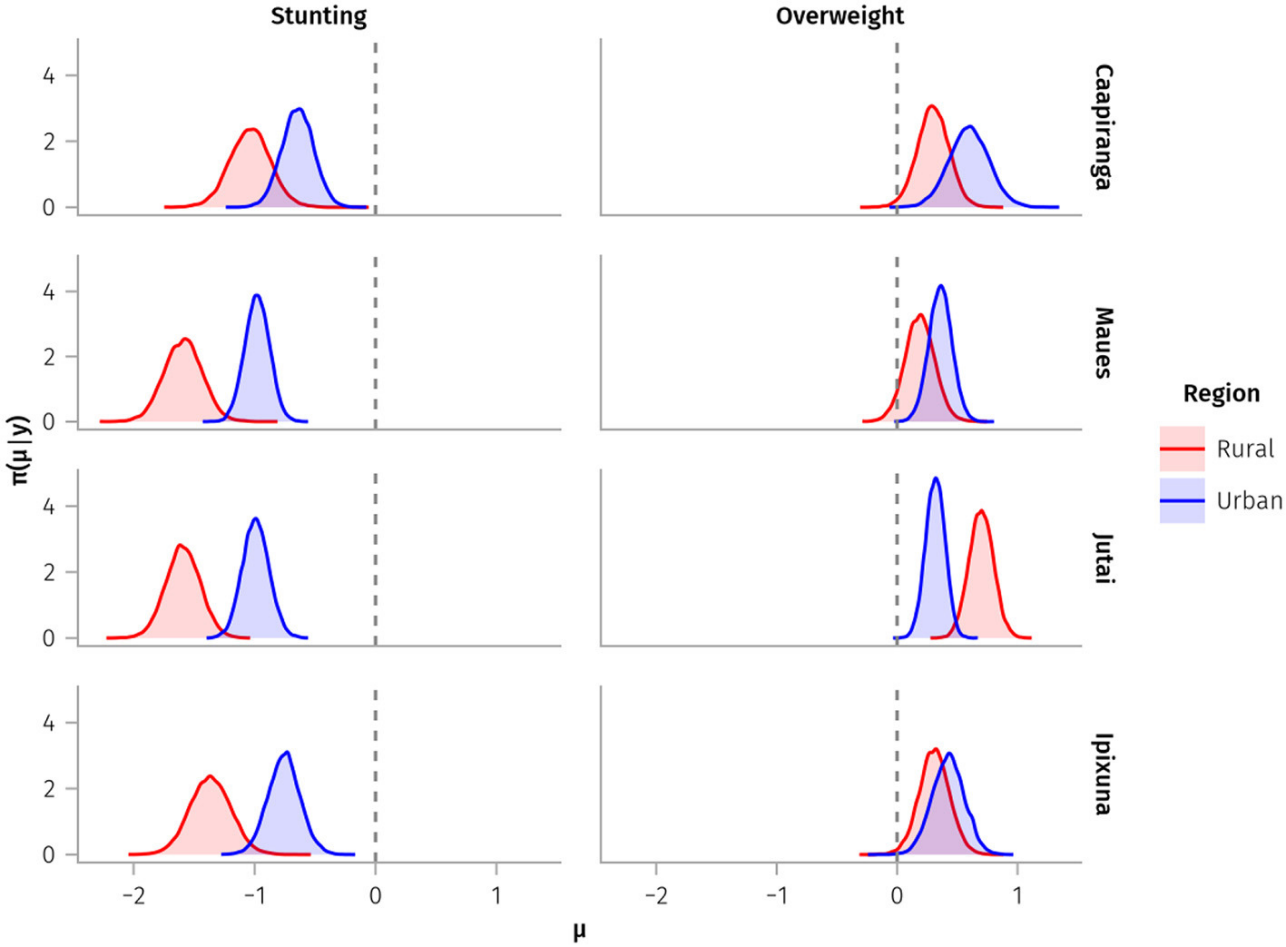
Posterior distributions of mean z-scores for stunting (height-for-age) and overweight (BMI-for-age) indicators (x-axes) for children under-5-years-old sampled from rural and urban sub-populations. Dashed vertical lines represent the median values of each indicator from the WHO reference population.

The posterior distributions of the means for the stunting indicator were below zero for both rural and urban sub-populations in all four municipalities (Figure 2), demonstrating an overall chronic nutritional disadvantage for children in this study. For all municipalities, there was a stronger rural tendency for stunted linear growth relative to urban sub-populations, in the sense that the distributions from rural sub-populations had lower mean height-for-age Z-scores compared to urban sub-populations. The apparent “urban advantage” was less pronounced in Caapiranga, seemingly due to lower rural stunting probability compared to other rural sub-populations. For overweight, the posterior distributions of all rural and urban sub-populations were substantially above zero (i.e., there was an overall tendency toward higher BMI-for-age). There was a slightly greater tendency toward overweight among urban sub-populations, apart from Jutai, where rural BMI-for-age z-scores were much higher than for urban children.

**Figure 3:**
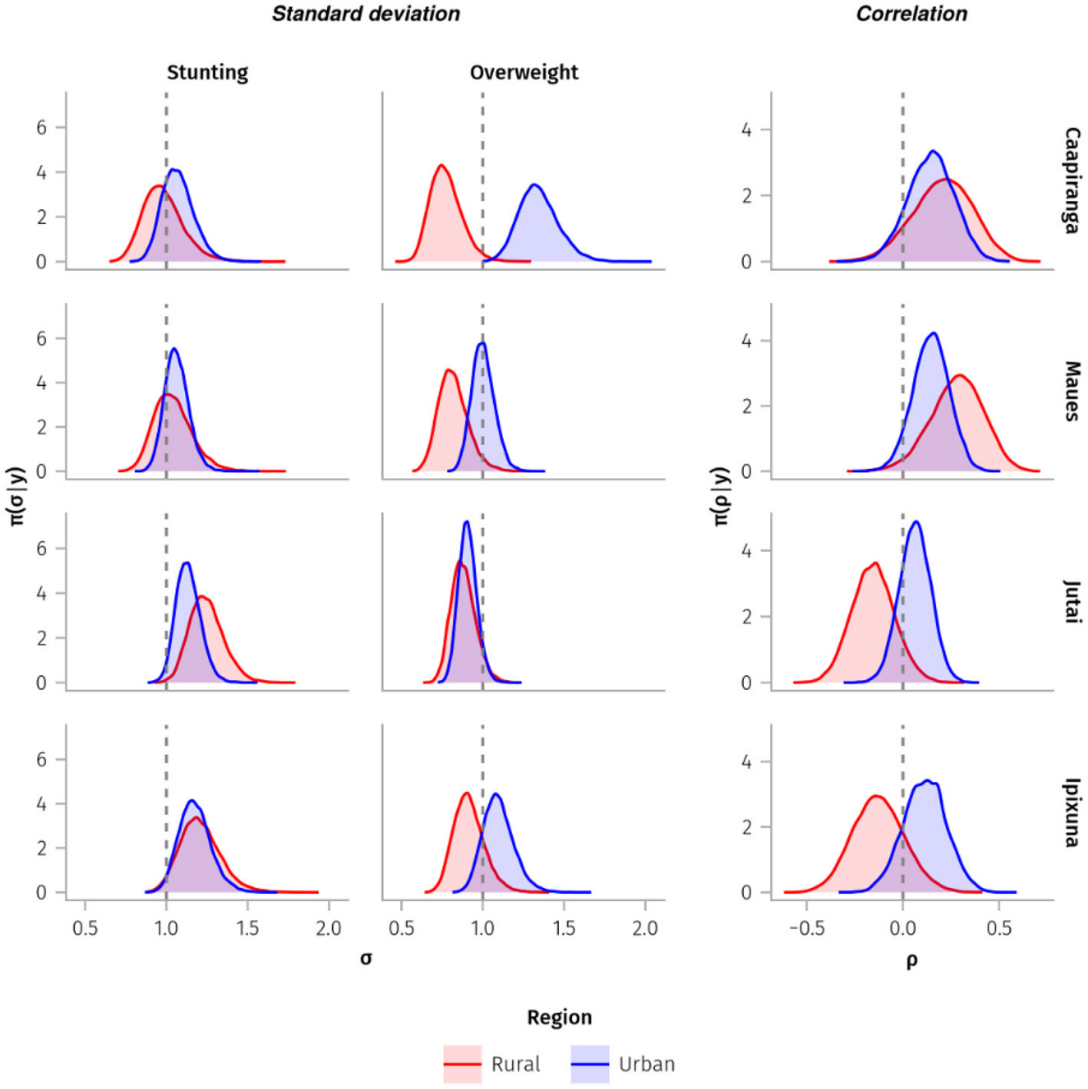
Posterior distributions of the standard deviations (σ) and correlations (ρ) (x-axes) of z-scores of height-for-age (stunting indicator) and BMI-for-age (overweight indicator) from children under-5-years old sampled in rural and urban sub-populations. Vertical dashed lines represent variability of 1 for the standard deviation sub-plots, and zero for the correlation sub-plots.

Most of the posterior distributions of the standard deviations for the height-for-age z-scores were above one among children in Ipixuna and Jutai, whereas the distributions of the standard deviations for Caapiranga and Maues children were clustered around one (Figure 3). Hence, variation in height-for-age was greater in the rural and urban sub-populations in Ipixuna and Jutai, and lower in Caapiranga and Maues. Within municipalities, we did not find substantial differences in stunting variability between rural and urban sub-populations. For overweight, the variability was lower in rural areas and was right-skewed distributed (with almost all variability ranging between 0.5 and 1.0 standard deviations), particularly in Caapiranga and Maues. Variability in overweight varied markedly among children in the four urban sub-populations and was notably high, above one, in Caapiranga.

Urban children tended to have positively correlated height-for-age and BMI-for-age z-scores, although this was less pronounced in urban Jutai (Figure 3). Rural patterns were more heterogenous; there was a positive correlation in these indicators for rural Caapiranga and Maues, whereas the correlations for rural children in Ipixuna and Jutai were mostly negative. The direction of the correlation between the indicators is important for estimating the prevalence of LDBM, as higher values will be observed when there is congruence between the direction of the bivariate distribution and the quadrant of interest. Accordingly, the exceedance probabilities and the estimated prevalence of LDBM were relatively high among rural children in Ipixuna and Jutai, compared to very low estimated prevalence for rural children in Caapiranga and Maues (Supplementary Table 2). There was a clear negative correlation between height-for-age and BMI-for-age z-scores in the rural areas of Ipixuna and Jutai, and a positive correlation in Caapiranga and Maues, regardless of whether the children were rural or urban residents (Supplementary Figure 1).

**Figure 4:**
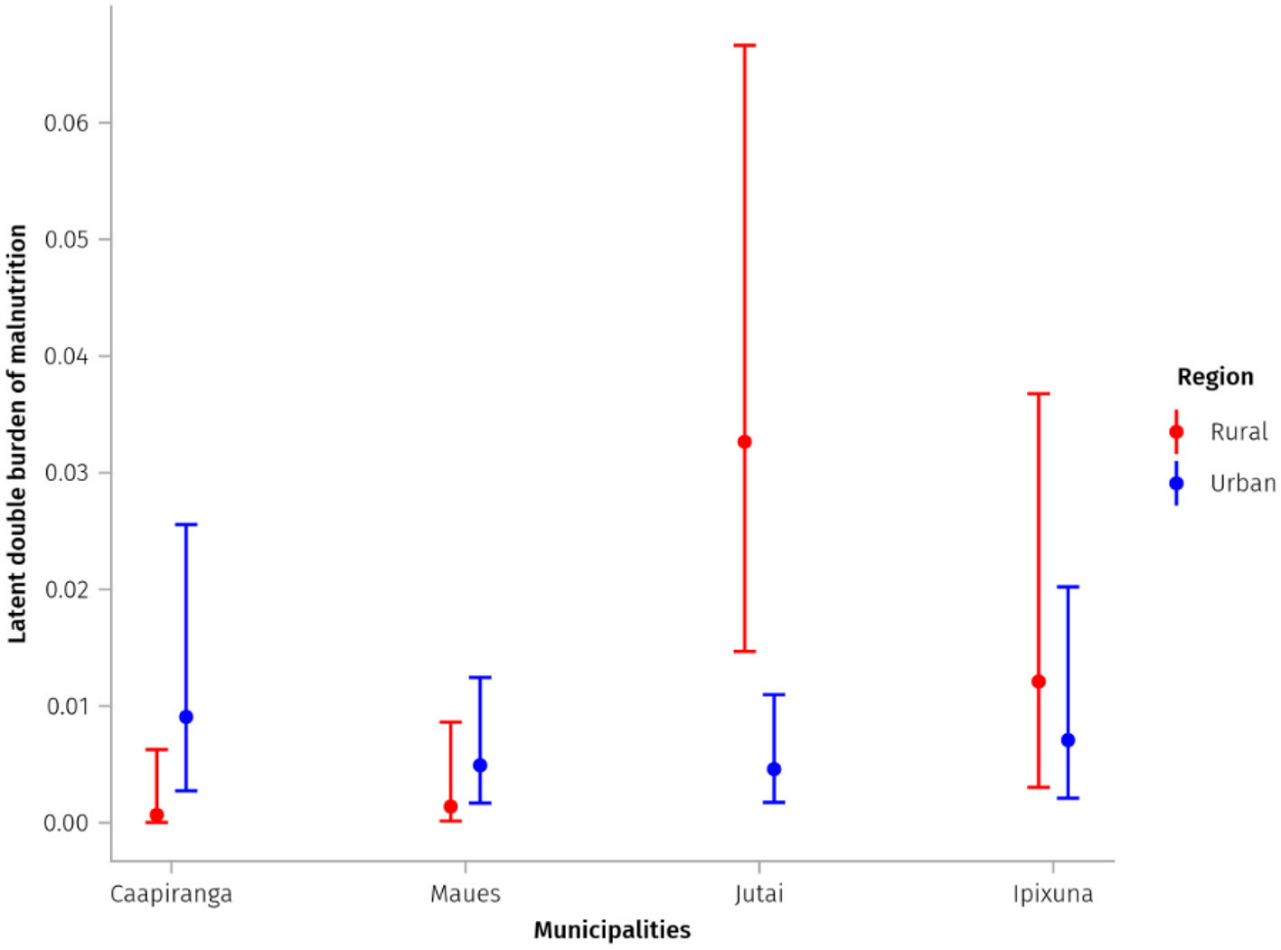
Estimated prevalence and quantile-based credible intervals (CI) of the Latent Double Burden of Malnutrition (overweight and stunting) in sampled rural and urban sub-populations.

The estimated prevalence of LDBM was highest in rural areas of Jutai (3.3%; CI: 1.5% to 6.7%) and Ipixuna (1.2%; CI: 0.3% to 3.8%), and the urban area of Caapiranga (0.9%; CI: 0.3% to 2.4%), and lower in other rural and urban areas (Table 2; Figure 4). The likelihood that LDBM prevalence exceeds 1.0% of children under-5-years-old was very high in rural Jutai (exceedance probability of 99.7%), rural Ipixuna (63.2%), and very low (< 2%) for the other two rural sub-populations. Exceedance probabilities also varied widely among urban sub-populations, from 6.7% in Maues to 41.2% in Caapiranga. The exceedance probability of LDBM prevalence being above 3.0% of children was high in rural Jutai (59.7%), and below 6% for all other sub-populations (Table 2). Consequently, in Jutai the prevalence of LDBM was significantly higher among rural children than among their urban counterparts, whereas we did not find evidence of meaningful rural-urban differences (i.e., because credible intervals overlapped) in other municipalities (Figure 4). The prevalence of LDBM was relatively similar across urban sub-populations, whereas rural prevalence varied more, being significantly higher in Jutai than in Maues or Caapiranga (Figure 4).

**Table 2:** Exceedance probabilities^*^ (Pr), median, lower limits (LL) and upper limits (UL) of the credible intervals for the prevalence of the latent double burden of malnutrition (overweight and stunting) among children under 5-years-old in rural and urban areas of Amazonas State, Brazil.

**Area**	**Municipality**	**Pr (p > 0.01)**	**Pr (p > 0.03)**	**Median**	**LL**	**UL**
Rural	Caapiranga	0.007	0.0	0.001	0.0	0.006
Rural	Maues	0.016	0.0	0.001	0.0	0.009
Rural	Jutai	0.997	0.587	0.033	0.015	0.067
Rural	Ipixuna	0.618	0.062	0.012	0.003	0.037
Urban	Caapiranga	0.433	0.011	0.009	0.003	0.026
Urban	Maues	0.07	0.0	0.005	0.002	0.012
Urban	Jutai	0.042	0.0	0.005	0.002	0.011
Urban	Ipixuna	0.264	0.002	0.007	0.002	0.02

^*^Exceedance probabilities (pr) surpassing 1 or 3%.

## Discussion

This study is the first, to our knowledge, to estimate the DBM at the individual level among rural and urban children using Bayesian-inference latent modeling. Our approach was designed to improve latent prevalence estimates for low prevalence phenomena, such as DBM. We applied our novel analytical technique to a unique dataset of similar-aged children randomly sampled within remote, river-dependent municipalities in the Brazilian Amazon; an under-studied and historically marginalized population which is vulnerable to the effects of the climate crisis, and other shocks and stressors (36, 37).

In our study, latent DBM (LDBM) at the individual level was at low prevalence (1.2% or below) in the sampled sub-populations, apart from one (rural Jutai), at 3.3% (CI: 1.5% to 6.7%) of children. A meta-analysis using a frequentist approach to report individual-level stunting and overweight DBM among children under-5-years-old found a mean prevalence of just 2.3% in low-income countries and 2.7% in middle-income countries (38). Tzioumis et al. (38) used data from Demographic and Health Surveys in 36 countries, yet their study populations may be more similar to ours than to that of the National Study of Food and Nutrition (ENANI) of nearly 15,000 Brazilian children (39). In Amazonas State (with 62 municipalities) the ENANI sample included only 42 children from metropolitan Manaus and a handful from two proximate road-connected municipalities. In Amazonas, the coverage and implementation of universal healthcare (e.g., adequate prenatal care), food and nutrition policies (e.g., adequate school meals, municipal Food Security Councils), and social protections (e.g., Maternity Pay) can be relatively weak outside of Manaus (25, 40, 41). Latent modelling is well-suited for studies with restricted sample sizes, such as with hard-to-reach or modest-sized focal populations.

Our latent models draw on observed variability in stunting and overweight indicators in small-samples and demonstrate that DBM prevalence risks can be above zero for particular sub-populations, even if no DBM cases are recorded based on frequentist classification. For instance, in the rural sub-populations of Caapiranga and Maues (the less remote municipalities in this study), where the randomly sampled households had fewer children under-5-years-old, there was a positive correlation between height-for-age Z-scores and BMI-for-age Z-scores. For those sub–populations, there were zero cases of DBM using the frequentist approach yet the estimated prevalences of LDBM were different from zero. Moreover, using our Bayesian approach it was possible to estimate credible intervals, parameters such as mean, median, and exceedance probabilities In the two extremely remote municipalities, Ipixuna and Jutai, the z-score correlation was reversed; short height-for-age rural children tended to be overweight, and rural LDBM prevalence was higher than in the less-remote municipalities. This may reflect that healthcare access, sanitation coverage, employment opportunities and income, state-led food and nutrition security interventions, and other social determinants of health (42) are worse in more remote parts of Amazonia (25).

Despite the wide credible intervals, we estimated higher point prevalence of LDBM in Jutai and Ipixuna's rural areas compared to their urban centres, consistent with existing research in LMICs and the well-established notion of “urban advantage” in health and nutrition. Tzioumis et al. (38) found lower prevalence of stunting and overweight coexistence among urban children (1.1%) compared to their rural counterparts (2.0%). In Brazil, DBM prevalence at the individual level is estimated to be 1.0% among the general population of children aged 5-to-11-years-old (43). A survey of children under-5-years-old in Kenya observed a higher occurrence of stunting and overweight in the rural zone in comparison to urban zone for both sexes (44). The occurrence of individual-level stunting and overweight DBM in children under-5-years-old in two districts in South Africa had a prevalence of 5.7%, with no significant difference between urban and rural areas (45).

We found evidence of an emerging malnutrition concern in rural Jutai, where the exceedance probabilities of LDBM being above 1% and 3% of children were very high (99% and 60%, respectively). The geographical locations of the rural communities sampled in Jutai may explain this sub-population's higher DBM prevalence. The town of Jutai and some of the surrounding rural communities we sampled are located on the banks of the Solimões River, between the regional urban hubs of Tabatinga and Tefé. Towns on this stretch of river have relatively good access to passenger-cargo boats (39), enabling surrounding rural communities to access obesogenic food products (46–49), including ultra-processed foods. Infant formula milk products may be reaching these communities through floating markets, and competing with breastfeeding. This is problematic because breastfeeding is protective against stunting and overweight (50, 51). This may partly explain the greater shift to the right in the overweight curve of children in rural Jutai, compared to other rural sub-populations.

Although we did not find evidence of significant differences between the credible intervals of LDBM prevalence across the four sampled urban areas, no null prevalences were generated, and the point estimate of LDBM in Caapiranga was slightly higher than in the other urban sub-populations. Furthermore, interval estimates indicate that this value could approach 2.3%, and the highest probability of this prevalence exceeding 1% in the urban area was in Caapiranga, at about 41%. We cannot fully explain the differences in the LDBM among towns. Nonetheless, potential explanations include the spatial proximity of Caapiranga to the metropolis of Manaus (159 km travel distance), which could facilitate access to ultra-processed products, usually high in fat, sugar, or sodium and associated with overweight/obesity (43, 52–54). Other possibilities include the influence of socioeconomic variables not evaluated in our study such as maternal education, family size, maternal height, and birth weight, child's diarrhea and household sanitation (55, 56).

Our results demonstrate that the precision of LDBM estimates may vary depending on sample size, the variability of posterior distributions, and the congruence of these parameters with the probability that a given child's height-for-age Z-score and BMI-for-age Z-score are simultaneously below −2 and >2 standard deviations, respectively (upper-left quadrants in the subplots of Supplementary Figure 2). The credible intervals for LDBM prevalence were relatively wide for two urban sub-populations (Caapiranga and Ipixuna), which also had smaller sample sizes and asymmetric, right-shifted variation in the malnutrition indicators compared to the other two urban sub-populations. Interestingly, although more rural children were sampled in Ipixuna and Jutai than in Caapiranga and Maues, credible intervals were much wider for the former two than the latter, possibly related to the negative correlation pattern observed in both urban sub-populations, including a substantial number of marginal values for DBM. Overall, our results show the limitations of traditional frequentist approaches for assessing low-prevalence malnutrition outcomes in relatively small samples. Restricted sample sizes are a common challenge for studies involving specific, and geographically hard-to-reach population groups such as indigenous peoples and other traditional forest-dwelling peoples in Amazonia (23, 57, 58). Our findings demonstrate that these challenges can be partially overcome through the application of Bayesian latent models that account for marginal values rather than only considering observed cases for point prevalence estimates, and instead using credible intervals and exceedance probabilities. Even using Bayesian latent models, however, further stratifying our modestly-sized sample by age group (for example) would tend to increase the credible intervals, limiting the interpretation of results.

Our study, combined with evidence from other LMICs, suggests that children from marginalized populations—whether living in rural or urban areas—are susceptible to stunting and overweight DBM (2, 4, 59). Poor dietary nutrition in terms of both quality and quantity is one of the possible mechanisms for the co-occurrence of DBM (2). The replacement of traditional dietary patterns with ultra-processed products, a phenomenon that has intensified in LMICs like Brazil, may be a crucial factor for the increase in DBM (21, 23, 52, 54, 57). It is no coincidence that national surveys point to socioeconomically vulnerable population strata as the most susceptible to the rising trend in the consumption of ultra-processed foods among Brazilians (60). Therefore, interventions aimed at mitigating DBM should consider contextual determinants of diet (9, 23, 57, 61, 62).

Our study represents an advance by applying Bayesian latent models to compare different contexts of DBM emergence at the individual level among children living in remote areas of Amazonia. Geographically specific studies into under-researched populations are important because the literature on DBM is mainly limited to research assessing DBM in terms of co-occurrence at the community or household-level (7, 38). Furthermore, although certain credible intervals of our estimates were relatively wide, possibly due to sample size, when compared with results from national surveys, we emphasize the need for analytical approaches that allow for the assessment of low-occurrence outcomes in specific groups with restricted population sizes. Aggregating population data at the national, state, or municipal level may obscure health inequities and render invisible the health inequities experienced by marginalized river-dwelling populations in remote parts of Amazonia.

Using Bayesian latent models may be useful for research or monitoring into other low-occurrence health or nutrition conditions at the population level, especially for initiatives lacking the resources of national- or international-scale studies and related sample sizes. Nonetheless, we highlight some limitations with our study. Our Bayesian approach using latent models hinders comparability with case-based frequentist analyses of observed prevalence. Our approach also requires greater computational performance and more specialist programming skills relative to conventional statistical analyses. Nevertheless, Hossain et al.'s (63) study into DBM prevalence among reproductive-aged women found that a Bayesian approach like ours obtained more precise parameter estimates and robust conclusions compared with a classical analytical technique (logistic regression) for estimating the prevalence. However, the specificity of our studied population, while making it impossible to select larger and more diversified rural samples, also limits the definition of informative priors, which would facilitate more precise (narrower) credible intervals for estimated prevalence (63).

## Conclusion

Using latent Bayesian models, we assessed a malnutrition outcome of low prevalence (the coexistence of stunting and overweight in the same individual children) in relatively small sample sizes from remote towns and rural communities in Amazonia. Furthermore, we analyzed the latent risk of DBM in vulnerable and marginalized populations, where the health and nutrition status are often poorest and the public health policies tend to focus strictly on undernutrition. Our approach can help to obtain more accurate estimates of low prevalence outcomes, and support public health service provision for effectively monitoring DBM in LMICs, particularly in vulnerable and hidden populations.

## Data Availability

The raw data supporting the conclusions of this article will be made available by the authors, without undue reservation.
