# Supplementary material for: Estimating double burden of malnutrition among rural and urban children in Amazonia using Bayesian latent models
Source: Front Public Health. 2025 Mar 12;13:1481397. doi: 10.3389/fpubh.2025.1481397 (PMC11937108; doi:10.3389/fpubh.2025.1481397)
Supplement: Supplementary file 1 [file Table_1.docx]

**Supplementary Material**

Supplementary File 1

| Although the sampling was based on the 2010 public census, we identified that new residential areas had emerged in Jutai by 2015, based on consulting Google Earth imagery and ArcGIS 10.3 basemaps. We used these aerial images to estimate the number of households in these new residential areas. We then assigned a proportional number of target households to each area (e.g., if a new development polygon contained 200 of a town’s 5,000 households, it would receive 4% of the sampling effort (i.e., 8 target households). |
| --- |
| In rural areas, communities were selected to capture geographical variation in terms of distance to the nearest urban area and the type of natural environment (*varzea* floodplain or *terra firme* upland), given the association between these factors and health determinants, including public service provision, income and agricultural potential. The community selection process was informed by input from local stakeholders. In each municipality, we aimed to sample eight communities each season, without revisiting a community (i.e., 16 communities per municipality). In each season, we tried to select four communities located along the main river (e.g., River Solimoes, for Jutai) and four along one or the smallest sub-tributaries, with varying remoteness from the municipal urban area. |
| Of the eligible children, 14 were not at home, and the guardians of another ten children refused to allow the collection of a blood sample for hemoglobin measurement. In addition, three children were excluded from the analysis because they had physical or neurological problems. No children were excluded due to implausible anthropometric data. |

**Supplementary Figure 1.** Scatterplots showing the correlations between z-scores of height-for-age (stunting equal values <-2) and BMI-for-age (overweight equals values >2) for individual children in rural and urban areas of Amazonas State, Brazil. Each sub-plot presents data from a municipality consisting of random samples from the urban area (towns), and rural samples from surrounding settlements. In each subplot, the upper-left quadrant indicates cases of the double burden of malnutrition at the individual level following a frequentist approach.


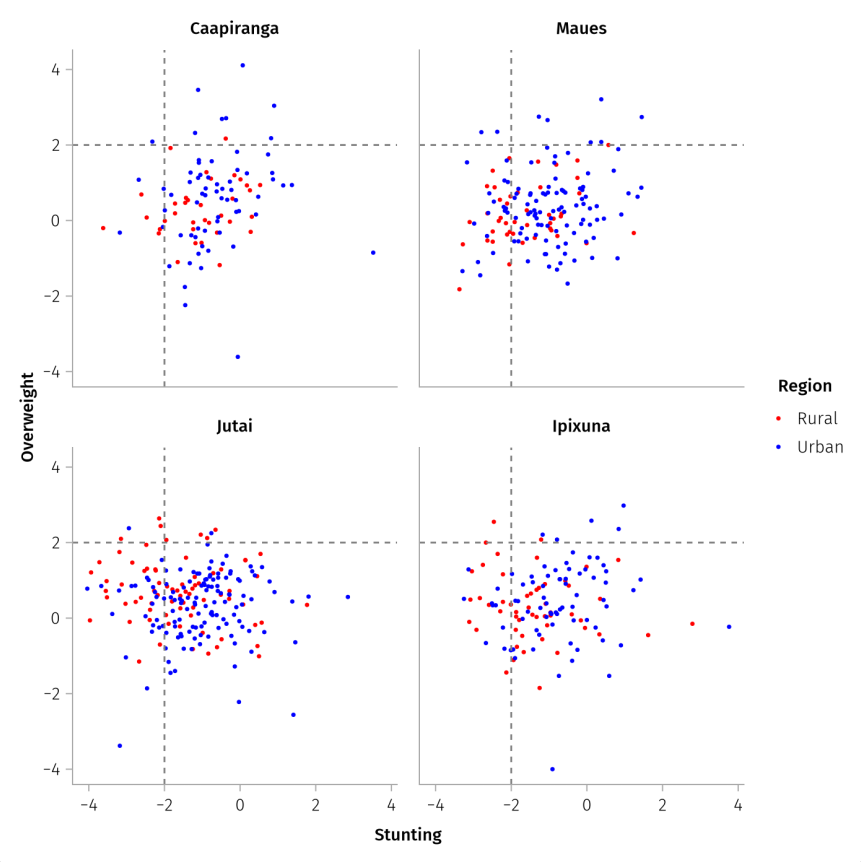


**Supplementary Table 1.** Likelihood ratio tests for the significance of household-level random effects by region type and municipality for z-scores of height-for-age and BMI-for-age. $D_{1}$ and $D_{2}$ represent the deviances of the models with and without random effects, respectively. Similarly, $AIC_{1}$ and $AIC_{2}$ are the Akaike Information Criteria for the models with and without random effects, respectively.

| *Z-score of height for age* | | |  |  |  |  |  |  |
| --- | --- | --- | --- | --- | --- | --- | --- | --- |
| **Region** | **Municipality** | **D₁** | **D₂** | **D₁-D₂** | **P-value** | **AIC₁** | **AIC₂** | **AIC₁-AIC₂** |
|  |  |  |  |  |  |  |  |  |
| Rural | Caapiranga | 94.69 | 93.25 | 1.44 | 0.23 | 98.69 | 99.25 | -0.56 |
| Rural | Maues | 124.99 | 123.69 | 1.31 | 0.25 | 128.99 | 129.69 | -0.69 |
| Rural | Jutai | 239.24 | 239.16 | 0.08 | 0.78 | 243.24 | 245.16 | -1.92 |
| Rural | Ipixuna | 163.80 | 163.80 | 0.00 | 1.00 | 167.80 | 169.80 | -2.00 |
| Urban | Caapiranga | 189.96 | 189.96 | 0.00 | 1.00 | 193.96 | 195.96 | -2.00 |
| Urban | Maues | 316.30 | 314.60 | 1.70 | 0.19 | 320.30 | 320.60 | -0.30 |
| Urban | Jutai | 400.50 | 394.17 | 6.34 | 0.01 | 404.50 | 400.17 | 4.34 |
| Urban | Ipixuna | 236.78 | 234.93 | 1.85 | 0.17 | 240.78 | 240.93 | -0.15 |
| *Z-score of BMI for age* | | |  |  |  |  |  |  |
| **Region** | **Municipality** | **D₁** | **D₂** | **D₁-D₂** | **P-value** | **AIC₁** | **AIC₂** | **AIC₁-AIC₂** |
| Rural | Caapiranga | 78.05 | 77.33 | 0.72 | 0.40 | 82.05 | 83.33 | -1.28 |
| Rural | Maues | 103.85 | 103.84 | 0.01 | 0.92 | 107.85 | 109.84 | -1.99 |
| Rural | Jutai | 187.77 | 186.89 | 0.88 | 0.35 | 191.77 | 192.89 | -1.12 |
| Rural | Ipixuna | 134.81 | 134.81 | 0.00 | 1.00 | 138.81 | 140.81 | -2.00 |
| Urban | Caapiranga | 219.50 | 219.50 | 0.00 | 1.00 | 223.50 | 225.50 | -2.00 |
| Urban | Maues | 303.81 | 303.81 | 0.00 | 1.00 | 307.81 | 309.81 | -2.00 |
| Urban | Jutai | 342.85 | 342.80 | 0.05 | 0.82 | 346.85 | 348.80 | -1.95 |
| Urban | Ipixuna | 225.20 | 221.07 | 4.13 | 0.04 | 229.20 | 227.07 | 2.13 |
